# Investigations into the Toxicological Properties of Aconitine

**Published:** 1869-04-01

**Authors:** D. Achscharumow


### *Investigations into the Toxicological Properties of Aconitine.*

BY D. ACHSCHARUMOW.

The author has used a solution of chloride of aconitine of one per cent. The poison was introduced under the skin, or carried into the stomach. The experiments were made upon frogs, pigeons and rabbits. In large doses aconitine induces death in a few minutes, after painful convulsions and the phenomena of asphyxia.

After the most minute dose, the following symptoms were observed:

Immediately after the poisoning, there was detected a retardation in the pulsations of the heart and in the respiration, together with diminished pressure in the vessels; respiration difficult and dyspnœic; cyanosis was rapidly developed; the temperature of the body was lowered.

Soon there occurred a persistent acceleration in the pulsations of the heart; respiration became more free; sanguineous pressure augmented; at the same time the phenomena of paralysis manifested themselves, and before they had acquired all their intensity, the activity of the heart again sank, and this organ was left distended passively with blood; circulation was arrested, convulsions supervened, and the animal died asphyxiated. After a very small dose the following symptoms appeared.

Salivation, difficult respiration, slight cyanosis, retardation of the pulsations of the heart, diminution of temperature, then, according to circumstances, the normal condition was restored, or the paralytic phenomena increased in intensity.

The author thus formulates his conclusions:

1st.—In poisoning by aconitine, death occurs by asphyxia, and by paralysis of the heart; paralysis which involves the motor ganglia of the heart.

2d.—The poison induces, first, excitation of the medulla-oblongata, which is transmitted to the vagi nerves.

3d.—The vagi nerves are paralyzed by a continuation of the excitation.

4th.—Paralysis of all the spinal motor nerves is induced, as well as of the motor centres of the heart, whose contractions are arrested. Soon the peripheral nervous extremities are entirely paralyzed, and voluntary movements become impossible. The muscular substance, however, remains intact.

5th.—The reflex functions of the medulla and the conductibility of the sensory fibres are not altered, sensibility is not involved.

6th.—The cervical portion of the great sympathetic is, probably, in a state of excitation.

7th.—Aconitine rapidly lowers the temperatnre of the body and the sanguineous pressure.

8th.—No cerebral disturbance has been recognized.

9th.—There is no local effect upon the pupil.

10th.—Applied upon the skin, aconitine does not traverse it, but remains inactive. Upon mucous surfaces it acts as a local irritant.

In a therapeutic point of view the author makes the following remarks:

1st.—Aconitine is not absorbed by the skin.

2d.—Its employment as a sedative agent is useless.

3d.—There can be no reason for employing it as a diuretic or a diaphoretic.

4th.—It may be employed internally in all morbid conditions, in which diminution of the temperature of the body, of the activity of the heart, or of sanguineous pressure, is indicated; as in simple or consecutive hypertrophy of the heart, in cerebral hyperæmias, in apoplexy, and generally in all hæmorrhages. Another indication for its employment is found in all the cases in which there is exaggeration of the action of the involuntary muscles, convulsions, tetanus, eclampsia.

